# Patients’ perceptions of 70-gene signature testing: commonly changing the initial inclination to undergo or forego chemotherapy and reducing decisional conflict

**DOI:** 10.1007/s10549-020-05683-6

**Published:** 2020-05-19

**Authors:** Julia E. C. van Steenhoven, Bianca M. den Dekker, Anne Kuijer, Paul J. van Diest, Peter Nieboer, Johanna M. Zuetenhorst, Alex L. Th. Imholz, Sabine Siesling, Thijs van Dalen

**Affiliations:** 1 Department of Surgery, Diakonessenhuis Utrecht, Utrecht, The Netherlands; 2 Department of Pathology, University Medical Center Utrecht, Utrecht University, Utrecht, The Netherlands; 3 Department of Radiology, University Medical Center Utrecht, Utrecht University, Utrecht, The Netherlands; 4 Department of Surgery, St. Antonius Hospital, Nieuwegein, The Netherlands; 5 Department of Medical Oncology, Wilhelmina Hospital Assen, Assen, The Netherlands; 6 Department of Medical Oncology, Franciscus Gasthuis Hospital, Rotterdam, The Netherlands; 7 Department of Medical Oncology, Deventer Hospital, Deventer, The Netherlands; 8 Department of Research, Netherlands Comprehensive Cancer Organisation, Utrecht, The Netherlands; 9 Department of Health Technology and Services Research, Technical Medical Centre, University of Twente, Enschede, The Netherlands

**Keywords:** Gene-expression profiling, Decision-making, Chemotherapy, Decisional conflict, Breast cancer

## Abstract

**Purpose:**

Little is known about the impact of 70-gene signature (70-GS) use on patients’ chemotherapy decision-making. The primary aim of this study was to evaluate the impact of 70-GS use on patients’ decisions to undergo chemotherapy. The perceived decision conflict during decision-making was a secondary objective of the study.

**Methods:**

Patients operated for estrogen receptor positive early breast cancer were asked to fill out a questionnaire probing their inclination to undergo chemotherapy before deployment of the 70-GS test. After disclosure of the 70-GS result patients were asked about their decision regarding chemotherapy. Patients’ decisional conflict was measured using the 16-item decisional conflict scale (DCS); scores < 25 are associated with a persuaded decision while a score > 37.5 implies that one feels unsure about a choice.

**Results:**

Between January 1th 2017 and December 31th 2018, 106 patients completed both questionnaires. Before deployment of the 70-GS, 58% of patients (n = 62) formulated a clear treatment preference, of whom 21 patients (34%) changed their opinion on treatment with chemotherapy following the 70-GS. The final decision regarding chemotherapy was in line with the 70-GS result in 90% of patients. The percentage of patients who felt unsure about their preference to be treated with chemotherapy decreased from 42 to 5% after disclosure of the 70-GS. The mean total DCS significantly decreased from pre-test to post-test from 35 to 23, irrespective of the risk estimate (p < 0.001).

**Conclusion:**

Deployment of the 70-GS changed patients’ inclination to undergo adjuvant chemotherapy in one third of patients and decreased patients’ decisional conflict.

**Electronic supplementary material:**

The online version of this article (10.1007/s10549-020-05683-6) contains supplementary material, which is available to authorized users.

## Introduction

In patients with early-stage breast cancer, adjuvant systemic therapy is administered to reduce the risk of cancer recurrence and to improve overall survival [1]. The advice to administer adjuvant chemotherapy (CT) is based on patients’ estimated risk of recurrence. Prognostic tools such as ‘Adjuvant!Online’ and ‘UK.Predict’ incorporate clinical and pathological risk factors to determine the recurrence risk and to guide clinical decision-making [2, 3]. Even with the aid of these algorithms individual risk assessment remains challenging as patients with comparable tumors may have different outcomes.

In general, patients with estrogen receptor positive (ER+), Her2 receptor negative (HER2-) breast cancer, have good prognosis and the incremental benefit of adding adjuvant CT to endocrine therapy (ET) is limited. However, some ER+ /HER2- patients have more aggressive tumor types who could benefit from CT. Over the past decades, focus has shifted towards optimal patient selection to determine in which patients the benefits of treatment with CT outweigh the negative effects. The use of adjuvant CT in ER+ /HER2- patients with no or limited axillary lymph node involvement has been decreasing during recent years [4, 5].

Several gene-expression profiles (GEP), such as the 70-gene signature (70-GS; MammaPrint) have been developed to provide more accurate risk assessment by classifying patients into two subgroups (low risk vs. high risk) on the basis of the risk of distant recurrence at 5 years and at 10 years[6–11]. Current breast cancer guidelines suggest the use of a validated GEP when there is doubt about the indication to administer CT in patients with ER+ invasive ductal carcinoma based on traditional prognostic factors [12, 13].

In a previous study, we assessed the impact of the 70-GS on CT-decisions in ER+ early breast cancer by asking physicians to formulate their advice before and after use of the 70-GS [14]. The results of that study showed that the 70-GS changed the physicians intended recommendation to administer CT in about half of the patients in line with the GEP result. Whereas the body of literature on the impact of GEP use on CT-decision making from a physicians’ perspective is growing [14–18], reports on patients’ perceptions on GEP use are scarce.

The primary aim of this prospective study was to evaluate the impact of 70-GS use on patients’ decisions to undergo adjuvant chemotherapy or not. Furthermore, we aimed to explore the perceived decisional conflict during decision-making and gain insight in patients’ understanding of 70-GS testing.

## Material and methods

### Study design and patients

This observational, prospective, questionnaire study was designed to assess the impact of 70-GS test on patients’ decision-making to undergo adjuvant CT or not. Patients for whom 70-GS test deployment was deemed indicated based on the prevailing national guideline [12] were eligible for participation. Exclusion criteria were a history of malignancy, the presence of distant metastasis, previous neo-adjuvant systemic treatment and inability to read or write Dutch. The study was approved by the medical ethics committee of the University Medical Center Utrecht and by institutional review boards of participating centers.

Between January 1 2017 and December 31 2018, patients were enrolled in nine participating centers in the Netherlands. The centers comprised both general non-teaching and teaching hospitals, located in the northern part and middle part of the country.

**Fig. 1:**
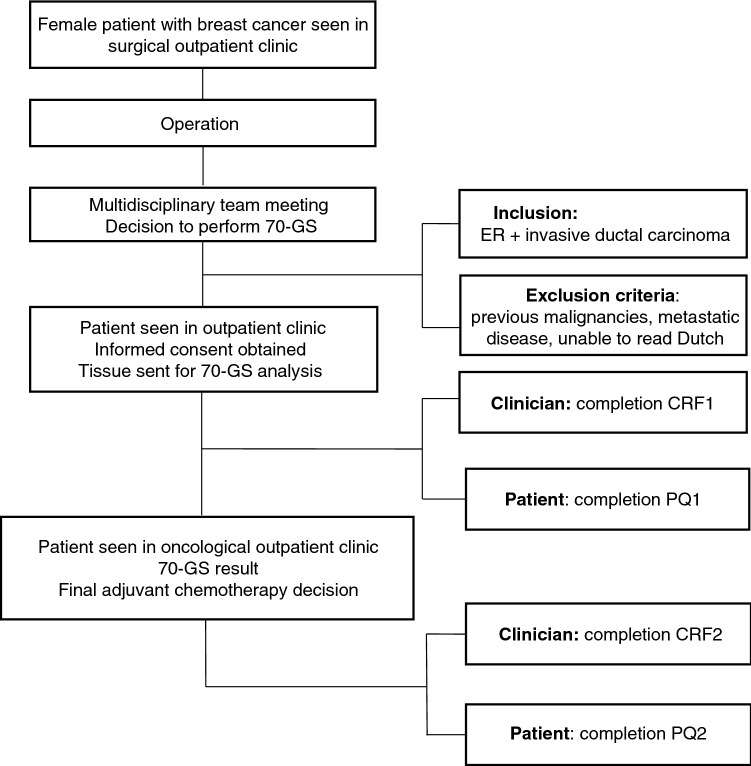
Flowchart of study inclusion between January 2017 and December 2019. *70-GS* 70-gene signature, *CRF1* clinical report form, *PQ* patient questionnaire, *ER* estrogen receptor

Figure 1 details the study flowchart. Eligible patients were identified during postoperative multidisciplinary team meetings based on the indication for 70-GS use to support the decision to administer adjuvant CT. Patients were informed about the study by their surgical oncologists or the medical oncologists following referral. Before deployment of the 70-GS test, informed consent was obtained from all participating patients. After enrollment and before deployment of the 70-GS, the treating physician completed the first clinical report form, in which information on clinicopathological characteristics and the preliminary CT recommendation to administer adjuvant CT, withhold adjuvant CT, or state uncertainty (i.e. depends on 70-GS result)—were registered. This CT recommendation was not disclosed to the patient. Simultaneously, an electronic questionnaire was sent to the patient. In this first questionnaire, information was obtained about the patients’ CT preference (to undergo CT-or-not, or ‘unsure’ when uncertain) without knowledge of the 70-GS. After completion of the clinical report form and submission of the first patient questionnaire, the tumor sample was sent for 70-GS analysis, and the result was disclosed to the oncologist within 10 working days. 70-GS analysis was carried out centrally by Agendia N.V. (Amsterdam, the Netherlands). A minimum tumor percentage of 30% in the tissue sample was required to obtain a valid result. After the 70-GS test result was disclosed, the treating physician reported the post-test CT recommendation and whether CT was actually administered in a second clinical report form. Patients received a second questionnaire regarding their final decision to undergo CT after receiving the 70-GS test, including survey items addressing the influence of the 70-GS test result on patients’ CT preference (Fig. 1).

### Decisional conflict

**Fig. 2:**
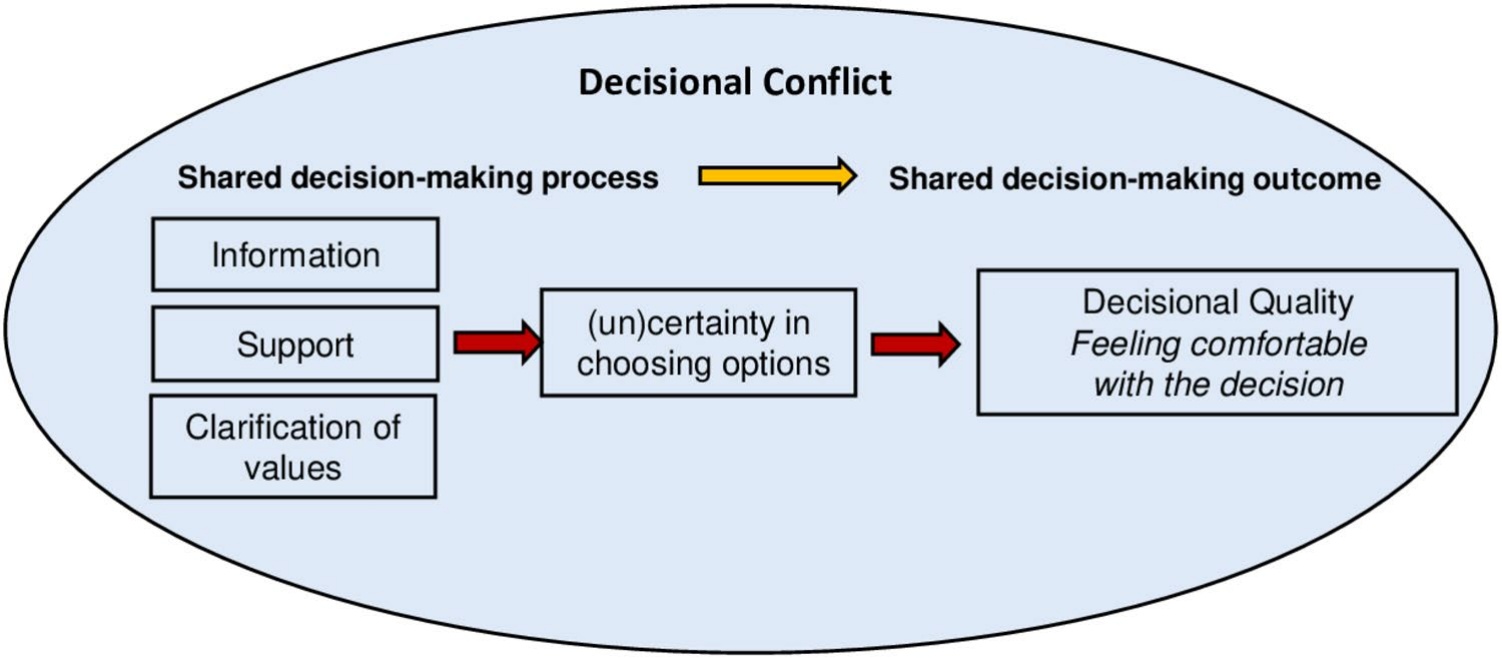
Decisional Conflict Model

Before and after disclosure of the 70-GS test result, patients were asked to fill out a decisional conflict scale (DCS). The DSC is a questionnaire widely used in health care studies of decision-making processes which measures the level of decisional conflict that patients experience while making treatment decisions and it has been validated in a breast cancer sample [19]. The DCS measures modifiable factors contributing to uncertainty in choosing options (e.g., support, information, clarity about personal values) and measures the eventual quality of the decision (Fig. 2). DCS scores range from 0 to 100, with 0 representing no decisional conflict and 100 reflecting the highest decisional conflict possible. According to this instrument, scores lower than 25 are associated with implementing decision, whereas scores exceeding 37.5 are associated with decision delay or feeling unsure about implementation [20]. Decisional conflict especially exists when a choice has to be made that involves uncertain risks or outcomes, which is the case in adjuvant therapy decision-making in cancer patients [19] and particularly in patients who receive systemic therapy in the adjuvant setting.

The DCS encompasses 16 items, each using a five-point response format (completely agree, agree, neither agree nor disagree, disagree, completely disagree). These items were categorized into five subscales measuring: being informed (extent to which one is informed about options, risks and benefit), values clarity (extent to which one feels clear about personal values and value trade-offs in the decision), support (extent to which one feels supported in making a choice), experiencing uncertainty (level of uncertainty in decision-making), and effective decision (extent to which one agrees their decision was informed, consistent with personal values and is likely to be implemented).

### Understanding of the 70-GS test result

In the second questionnaire, patients were queried about their understanding of the genomic test result using six knowledge questions. Patients received + 50 points when one of the following questions were answered with yes: ‘the 70-GS provides me information about the risk of distant metastases’ or ‘the 70-GS aids decisions about undergoing adjuvant CT’. Patient received −  50 points when one of the following questions were answered with yes: ‘the 70-GS gives me information about the presence of hereditary breast cancer’, ‘the 70-GS gives me information about the success of the operation’, ‘the 70-GS gives me information about my chance that adjuvant chemotherapy will be a success’ or ‘the 70-GS gives me information about my life expectancy. Scores of + 50 points or higher were associated with good understanding of the 70-GS test. Furthermore, patients were asked to report, to their personal opinion, their chance of breast cancer recurrence within 5 years. In order to identify characteristics associated with a patient’s understanding of the 70-GS, we obtained patient demographics including education level, employment status, family composition, county of birth and household income.

### End points

The primary end point of this study was defined as the percentage of patients for whom 70-GS use led to an altered adjuvant CT treatment preference (no CT, CT or CT unsure). Secondary endpoints included the change in mean DCS scores prior to and after deployment of the 70-GS, evaluation of patients’ understanding regarding 70-GS use, agreement on CT treatment preference between patients’ and oncologists’ recommendation and the adherence to the 70-GS test result. Patient characteristics associated with patients’ understanding of 70-GS testing were explored.

### Statistical analysis

The frequency of patients’ preferences to undergo CT was evaluated before and after use of the 70-GS. The change in mean total DCS scores before and after the 70-GS result and for the subscales were compared by a Wilcoxon signed-rank test. P-values ≤ 0.05 were considered statistically significant.

We calculated standardized effect sizes (*d)* by dividing the mean difference in DCS scores before and after use of the 70-GS by the pooled standard deviation (SD). Effect sizes around 0.2, 0.5 and 0.8 are considered small, medium, and large, respectively. Patients’ adherence to the 70-GS result was calculated by the sum of patients who adhered to the 70-GS result (i.e., prefer no CT in case of a low-risk profile and prefer administration of CT in case of a high-risk result) divided by the total number of patients. Agreement between patients’ preference and oncologists’ recommendation on CT treatment was evaluated. Logistic regression analysis was used to identify patient characteristics associated with a poor understanding of the 70-GS test.

## Results

### Patients

A total of 106 ER+ /HER2- negative breast cancer patients were enrolled in the study (median age 55 years). The majority of patients was surgically treated for unifocal, intermediate grade and T1c tumors. Fifty-nine percent of patients were diagnosed with pN0(i-/i +) disease, the remaining patients had (limited) axillary lymph node involvement (pNmi-pN1a). Eighty-seven percent of patients had been treated by breast conserving surgery (Table 1). The 70-GS stratified 77% of patients into the 70-GS genomic low-risk category.

**Table 1:** Clinical characteristics and demographics of estrogen receptor positive breast cancer patients (n = 106)

	%
Age (median, min–max)	55 years (34–70)
Type of surgery	
Breast conserving	87
Mastectomy	13
Progesterone receptor status	
Negative	3
Positive	97
Grade	
1	17
2	77
3	5
Unifocal tumor	
No	9
Yes	91
Tumor diameter in mm (median, min–max)	18 (8–48)
T-stage	
T1	62
T2	38
N-Stage	
N0	59
Nmi	15
N1a	25
Unknown	1
70-Gene signature test result	
High risk	23
Low risk	77
Education	
Primary school	4
High school diploma	27
Secondary vocational education	31
Higher professional education	23
University	15
Household income	
< €20.000	9
€20.000–€40.000	12
€40.000–€60.000	16
> €60.000	22
Prefer not to answer	41

### Patients’ adjuvant CT preference

**Fig. 3:**
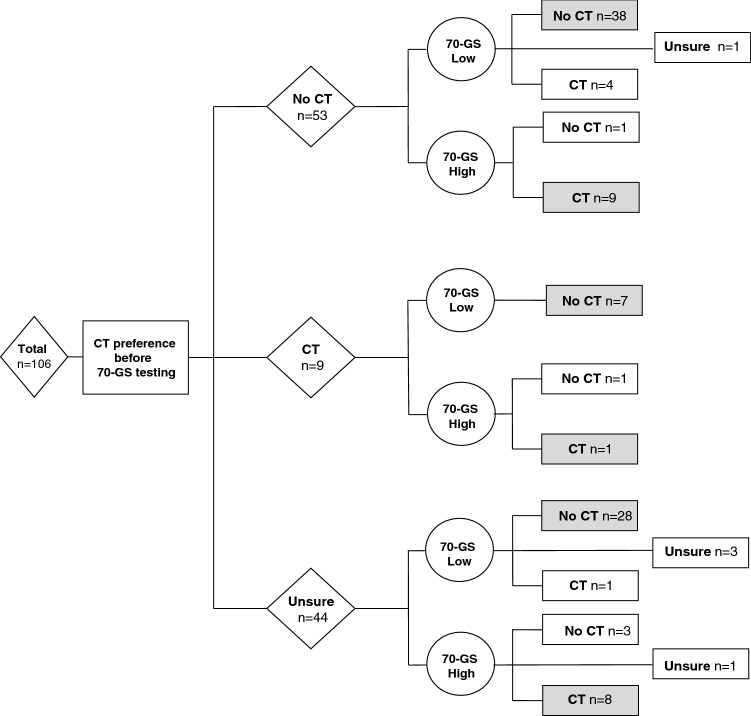
Flowchart describing patients’ inclination to undergo adjuvant CT before use of the 70-GS and the final decision to undergo adjuvant CT after the 70-GS result was disclosed to the patient. Patients in whom the final CT decision was in line with the 70-GS result are represented in gray. *CT* chemotherapy, *ER* estrogen receptor, *70-GS* 70-gene signature

Before deployment of the 70-GS, 58% of patients formulated a clear preference to undergo CT (n = 9) or not (n = 53), whereas 42% of patients felt unsure regarding this decision (Fig. 3). After disclosure of the 70-GS, 95% of patients formulated a clear decision and the percentage of patients who remained in doubt regarding their treatment decreased to 5% (Fig. 3). Of the 62 patients who formulated a clear preliminary decision before 70-GS deployment, 21 patients (34%) subsequently changed their opinion (from CT to no CT or vice versa). The overall agreement between the patients’ post-test CT preference and the 70-GS result was 90%: five patients eventually decided to have adjuvant CT despite having a low-risk test result and five patients preferred not to receive CT despite the presence of a 70-GS high-risk test result. Eighty percent of patients (n = 85) considered 70-GS a decisive factor regarding their final treatment plan.

### Decisional conflict

The mean total DCS-score before deployment of the 70-GS was 35 out of 100 and the mean total score decreased to 23 after disclosure of the 70-GS test result (p < 0.001, Table 2). We determined an effect size of 0.8 for the mean change in DCS following the 70-GS, which is considered large. The initial decisional conflict was highest in patients who preferred not to undergo CT (Tables 3 and 4). However, this subgroup of patients also showed the largest decrease in DCS when the final decision not to undergo CT was in line with the preliminary decision (mean change total DCS 14.0 points, Table 4). In the small subset of patients who remained unsure about CT, the mean total DCS-score increased (+ 8.0 points, DCS post 70-GS 41 out of 100, Table 4). In ten patients, the decision to undergo or forego adjuvant CT was not in line with the 70-GS result. In the five patients who eventually decided to undergo adjuvant CT despite having a low-risk test result, the post-test DCS was higher compared to the total group of patients (mean post-test DCS 32 vs. 23). In the five patients who decided to refrain from CT despite a high-risk test result, the mean total DCS decreased from 31 to 25 (data not shown).

**Table 2:** Changes in total decisional conflict scores and sub-scores with regard to the decision to undergo adjuvant chemotherapy for the total cohort of estrogen receptor positive breast cancer patients before and after being informed on the results of the 70-gene signature test (n = 106)

	Decisional conflict scores		
	Pre-test score	Post-test score	P-value*
Total	35	23	< 0.001
Sub-scores			
Informed score	28	19	< 0.001
Clarity score	35	22	< 0.001
Support score	25	22	0.26
Uncertainty score	47	28	< 0.001
Effective decision score	37	25	< 0.001

*P-values represent Wilcoxon signed-rank test

**Table 3:** Patients’ chemotherapy (CT) inclination prior to the 70-gene signature test (70-GS), baseline decisional conflict scale (DCS) scores and DCS scores after being informed on the 70-GS test results

Pre-70-GS CT inclination	Pre 70-GS DCS-score	70-GS result	Post 70-GS DCS-score
No CT (N = 53)	38	Low risk	25
		High risk	24
CT (N = 9)	29	Low risk	19
		High risk	25
CT Unsure (N = 44)	33	Low risk	21
		High risk	25
Total (N = 106)	35	Low risk	23
		High risk	24

**Table 4:** Patients’ chemotherapy (CT) inclination prior to the 70-gene signature (70-GS), baseline decisional conflict scale (DCS) scores and DCS scores after being informed on the 70-GS test results stratified by the patients’ final CT decision

Pre-70-GS CT inclination	Pre 70-GS DCS-score	Post 70-GS CT decision	Post 70-GS DCS-score
No CT (N = 53)	38	No CT	24
		CT	26
		Unsure	25
CT (N = 9)	29	No CT	19
		CT	25
		Unsure	–
CT Unsure (N = 44)	33	No CT	19
		CT	23
		Unsure	41
Total (N = 106)	35	No CT	22
		CT	25
		Unsure	38

Table 2 shows the difference in DCS for each subscore. Four out of five subscores significantly decreased after disclosure of the 70-GS test result. Only the ‘support’ score did not significantly decrease, albeit that the initial score was already low (25 at baseline), implying that patients felt supported regarding their decision-making throughout the decision-making process.

### Physicians’ adjuvant CT recommendation

Before deployment of the 70-GS, physicians refrained from recommending CT-or-not in 94% (n = 100) of patients. Physicians apparently preferred to await the 70-GS test result. In the remaining six patients, physicians did advise CT. The physician’s final treatment recommendation was in line with the 70-GS test result in 96% of patients (four patients were advised to receive adjuvant CT despite a low-risk test result). Agreement between patients’ final decision and the oncologists’ recommendation for treatment with CT was 92%.

### Patients’ understanding of the 70-GS

After disclosure of the 70-GS test result, 68% percent of patients understood that the 70-GS had provided information regarding their adjuvant CT benefit and 59% of patients understood that the test provided information regarding the risk for metastatic disease. See Supplementary Table 1. Thirteen percent answered that the test gave them information about their life expectancy and small proportions patients thought that the 70-GS had provided information regarding the success of the operation and that the test provided information about the presence of hereditary breast cancer. Furthermore, we observed a large variation in the patients’ self-reported risk of locoregional or distant recurrence at 5 years. For example, some 70-GS low-risk patients who were aware of this test result reported to have a 98% chance that their cancer would return within 5 years and some high-risk patients reported to have a 1% chance (data not shown).

In relation to patient characteristics, low education level of the patient (high school or less vs. at least some college) and older age (> 65 years vs. < 55 years) were negatively associated with a correct understanding of the 70-GS (OR 0.19 95%CI 0.03–0.84 and OR 0.25 95%CI 0.07–0.86, respectively). Other patient demographics (household income, employment status, country of birth, family composition) failed to identify any significant correlations (data not shown). Understanding of the 70-GS did not differ between patients with a low or high-risk 70-GS test result or between patients with a high or low DCS (data not shown).

## Discussion

In this prospective study in breast cancer patients in whom the 70-GS was deployed, one third of patients changed their intended decision to undergo adjuvant CT following disclosure of the test result. Deployment of the 70-GS into the decision-making regarding CT was associated with a significant decrease in decisional conflict and a significant increase in the proportion of patients that felt sure about their decision. Low education level and older age were negatively associated with a correct understanding of the 70-GS test.

Thirty-four percent of the patients changed their mind after disclosure of the 70-GS test result. While most tests expressed a genomic low risk, twenty five percent of the patients who initially felt they should not undergo CT eventually decided that they would receive CT and eight of the nine patients refrained from chemotherapy despite an initial preference for it. In addition, we found that the percentage of patients who were initially unsure about treatment with adjuvant CT decreased from 42 to 5% after use of the 70-GS. These results complement previous findings evaluating the impact of GEP use on the shared decision-making process regarding adjuvant CT [21–24]. In a large prospective study conducted by Levine et al. the impact of Oncotype Dx on the patient’s CT preference was assessed in the same category of ER + /HER2- breast cancer patients and they reported a comparable 31% change in the patient’s CT treatment choice following Oncotype Dx. Their study also reported a similar proportion of patients (42%) feeling initially unsure about their CT choice [21]. Most patients downgraded their choice from CT to no CT following Oncotype Dx. Comparable results regarding the impact of genomic testing in the clinical decision-making process of the patient following EndoPredict have been reported as well [25]. The use of Endopredict led to an altered CT preference in 37% of patients, of which half of the patients upgraded their choice to CT and half of the patients downgraded their choice to endocrine therapy. We observed high adherence rates of patients and clinicians to the 70-GS test result which is in line with other studies [26, 27]. This finding supports a previous study evaluating how patients valued GEP testing in their treatment decision. Many of these patients described the test as an element that empowered them, allowed them to feel confident in their decision, and in many cases, rescued them from unnecessary CT [28].

Another important finding of this study was the reduction in decisional conflict following use of the 70-GS. A mean post-test DCS of 23 implies that patients were convinced of their choice. The magnitude of the reduction measured by the effect size (*d* = *0.8*) outpaced the effect size what is considered a clinically important and meaningful difference (*d* = *0.40*) for this tool [19, 20]. Our findings are in line with previous studies who also found a significant reduction in DCS and a substantial decrease in patient anxiety too [18, 21, 25–28]. In the present study, the decrease in DCS was influenced by differences in the patients’ pre- and post-test CT treatment preferences. Before the test was deployed, patients who intended not to undergo CT felt most uncertain about their decision, while the post-test score was the lowest in those in whom treatment was downgraded. Decisional conflict was the highest in the small group of patients in whom uncertainty remained despite the use of this test and in patients who choose to undergo CT despite a low-risk test result.

Exploring the patients’ understanding of the 70-GS, we observed that most patients (68%) were aware of the purpose of the test, i.e., they knew that the test provided information regarding the benefit of CT. At the same time, a substantial proportion of patients (41%) did not understand that this information also implied a higher or lower risk of developing distant recurrence. The lack of knowledge of GEP testing is also illustrated another study in which patients tended to overestimate the truth-value of the test based on misperception on its validity [29]. Given the increased use of multigene assays to guide systemic treatment decisions [4], it is of importance to identify knowledge gaps in patients’ understanding regarding the clinical implication of a GEP-test. Despite the large confidence intervals as a result of limited sample size, our findings suggested that low education level and older age were associated with poor understanding of the 70-GS test. These findings should stimulate clinicians to optimize their communication strategies in order to explain the purpose of the test, adjusted to the education level and age of the patient. A previous study reported that oncologists considered explaining GEP testing to patients in a simple way, but, paradoxically, they remained uncertain about patients’ understanding of genomic testing [30].

There are some limitations of this study. First, the number of patients within the study cohort is limited and information regarding the total number of eligible patients within the institutions that were not invited to participate or were not enrolled in the study is lacking. The limited number of patients precludes firm conclusions, particularly regarding the DCS variation and the identification of factors associated with a poor understanding of 70-GS testing. Furthermore, while we observed an important decrease in the proportion of patients who felt unsure about whether or not to undergo CT following the 70-GS test, this could well be the result of the fact that these patients were aware that the 70-GS would provide additional information regarding the effect of the therapy. In addition, in this study we cannot correct for the effect of time on the decrease in decisional conflict, since contemplation during a cooling-off of 10–14 days may well have an effect on the perceived decisional conflict. Ideally, we would have used a control group of breast cancer patients in whom the 70-GS was not applied to compare the difference in decisional conflict within these two groups. On the other hand, our study examined the 70-GS associated treatment preference together with the effect on patients’ decision conflict. The study design and population best mimics routine practice.

In conclusion, use of the 70-GS changed the patient-intended preference to undergo adjuvant CT in one third of patients and helped patients to feel more confident about their adjuvant CT choice. Deployment of the 70-GS was associated with a significant and clinically relevant decrease in patients’ decisional conflict.

## Electronic supplementary material

Below is the link to the electronic supplementary material.

Supplementary file1 (DOCX 28 kb)
