# Supplementary material for: Patients’ perceptions of 70-gene signature testing: commonly changing the initial inclination to undergo or forego chemotherapy and reducing decisional conflict
Source: Breast Cancer Res Treat. 2020 May 19;182(1):107–15. doi: 10.1007/s10549-020-05683-6 (PMC7275022; doi:10.1007/s10549-020-05683-6)
Supplement: Supplementary file 1 — Supplementary file1 (DOCX 28 kb) [file 10549_2020_5683_MOESM1_ESM.docx]

**Supplementary Table 1**. Responses to questions assessing patients’ understanding of the 70-GS test.

| Questions | Response  Total N=106 | |
| --- | --- | --- |
|  | Yes N (%) | No N (%) |
| The 70-GS gives me information about the presence of hereditary breast cancer | 5 (5%) | **101 (95%)** |
| The 70-GS gives me information about the success of the operation | 9 (8%) | **97 (92%)** |
| The 70-GS gives me information about the risk of distant metastases | **63 (59%)** | 43 (41%)_ |
| The 70-GS gives me information about the benefit of adjuvant chemotherapy | **72 (68%)** | 34 (32%) |
| The 70-GS gives me information about my chance that adjuvant chemotherapy will be a success | 10 (9%) | **96 (91%)** |
| The 70-GS gives me information about my life expectancy | 14 (13%) | **92 (87%)** |
| Abbreviations: 70-GS, 70-gene signature. Numbers in bold represent a correct response. | | |
